# Tissue engineering the cancer microenvironment—challenges and opportunities

**DOI:** 10.1007/s12551-018-0466-8

**Published:** 2018-11-08

**Authors:** Vassilis Papalazarou, Manuel Salmeron-Sanchez, Laura M. Machesky

**Affiliations:** 1 CRUK Beatson Institute for Cancer Research and Institute of cancer Sciences, University of Glasgow, Garscube Campus, Switchback Road, Bearsden, Glasgow, G61 1BD UK; 2 The Centre for the Cellular Microenvironment, University of Glasgow, Glasgow, G12 8QQ UK

**Keywords:** Mechanosensing, Cancer microenvironment, Extracellular matrix, Adhesion, Cell migration, Cytoskeleton, Motility, Hydrogels

## Abstract

Mechanosensing is increasingly recognised as important for tumour progression. Tumours become stiff and the forces that normally balance in the healthy organism break down and become imbalanced, leading to increases in migration, invasion and metastatic dissemination. Here, we review recent advances in our understanding of how extracellular matrix properties, such as stiffness, viscoelasticity and architecture control cell behaviour. In addition, we discuss how the tumour microenvironment can be modelled in vitro, capturing these mechanical aspects, to better understand and develop therapies against tumour spread. We argue that by gaining a better understanding of the microenvironment and the mechanical forces that govern tumour dynamics, we can make advances in combatting cancer dormancy, recurrence and metastasis.

**Electronic supplementary material:**

The online version of this article (10.1007/s12551-018-0466-8) contains supplementary material, which is available to authorized users.

## Introduction

One of the biggest challenges in the treatment of cancer is to develop better ways to predict, detect and eradicate the spread of tumour cells to distant tissues. Cancer cells interact dynamically with their surrounding environment and not only remodel the nearby extracellular matrix but also affect immune cell infiltration, local fibroblasts and distant tissues. Pancreatic ductal adenocarcinoma (PDAC) provides an example of a cancer that is characterised by aggression fuelled by the microenvironment. PDAC tumours are often highly fibrotic with excessive deposition of extracellular matrix (ECM) molecules, including fibrillar collagen. Excess matrix deposition not only contributes to the aggressiveness of the malignancy but also poses major constraints on the delivery of chemotherapeutic reagents to the tumour (Kleeff et al. 2016). This so-called desmoplastic, collagen-rich stroma has been the target of recent therapeutic intervention strategies, with attempts to ‘normalise’ the stroma to allow better access of chemotherapy or immunotherapy, reviewed in (Vennin et al. 2018). However, the role of this dense matrix is complex and it remains poorly understood which stromal aspects prevent or promote tumorigenesis. Unfortunately, attempts to ablate the matrix have so far not led to patient benefit and may even cause harm (reviewed in (Neesse et al. 2015)). We will explore how recent developments in bioengineering might improve modelling the interactions between tumour cells and the microenvironment to hopefully improve development of new therapies against metastasis and recurrence (Table 1).

**Table 1:** Summary of processes affected by mechanical properties of the environment and associated references

Mechanical properties	Processes affected	Processes affected	References
Stiffness	Mechanosensing	Yap/Taz, integrin signalling, RTK signalling, Wnt signalling, Piezo, GTPases	Aragona et al. 2013; Diamantopoulou et al. 2017; Dupont et al. 2011; Halder et al. 2012; Lin et al. 2015; Panciera et al. 2017; Zanconato et al. 2016
Viscoelasticity	Mechanosensing	Yap/Taz, integrin signalling, GTPases	Bennett et al. 2018; Chaudhuri 2017; Chen et al. 2015; Wang et al. 2018a, b
Architecture			
Fibre alignment	Migration direction cell density	Actin dynamics, adhesion	Ahmadzadeh et al. 2017; Chaudhuri et al. 2014; Conklin et al. 2018; Drifka et al. 2016; Fraley et al. 2015; Mouw et al. 2014; Nuhn et al. 2018; Patel et al. 2018; Yang et al. 2017
Matrix geometry-pore size	Nuclear squeezing, rupture genomic instability	Nesprin/SUN proteins DNA damage	Bennett et al. 2017; Denais et al. 2016; Elosegui-Artola et al. 2017; Harada et al. 2014; Isermann and Lammerding 2017; Lautscham et al. 2015; Lombardi et al. 2011; Rothballer et al. 2013; Wolf et al. 2013; Woroniuk et al. 2018
Topography curvature	Curvature sensing cytoskeleton/signalling?	BAR domain proteins	Harada et al. 2014; Chen et al. 2012; Heath and Insall 2008

Epithelial tumours are a complex mixture of cancer cells, normal cells and extracellular matrix. Tumours disrupt organ structure and break the normal rules of organisation, growth control and boundary respect. They harbour fibroblasts and immune cells, as well as their own vasculature and lymphatic vessels. Tumours are inflamed and have been described as wounds that never heal, having lost normal signals that allow tissues to maintain their structural and biological framework (Dvorak 2015). In particular, wound healing is a multiparametric process of stochastic events including cell infiltration, ECM deposition and remodelling, where mechanical regulation restores tissue homeostasis and architecture. However, loss of mechanical checkpoints could facilitate neoplasm generation and growth. In addition, tumour vasculature is tortuous and leaky, giving access to tumour cells and preventing oxygen and nutrient delivery in areas of the tumour. When combined with the excessive mutation rates and genomic instability of cancer cells, the aforementioned parameters can drive tumours to break away from their primary site and metastasise. Thus, a thorough understanding of mechanical forces that organise normal and malignant tissues is essential. We argue that recent advances in bioengineering can make exciting contributions to combatting tumour progression and dissemination by revealing how forces shape tissues and tumours.

**Fig. 1:**
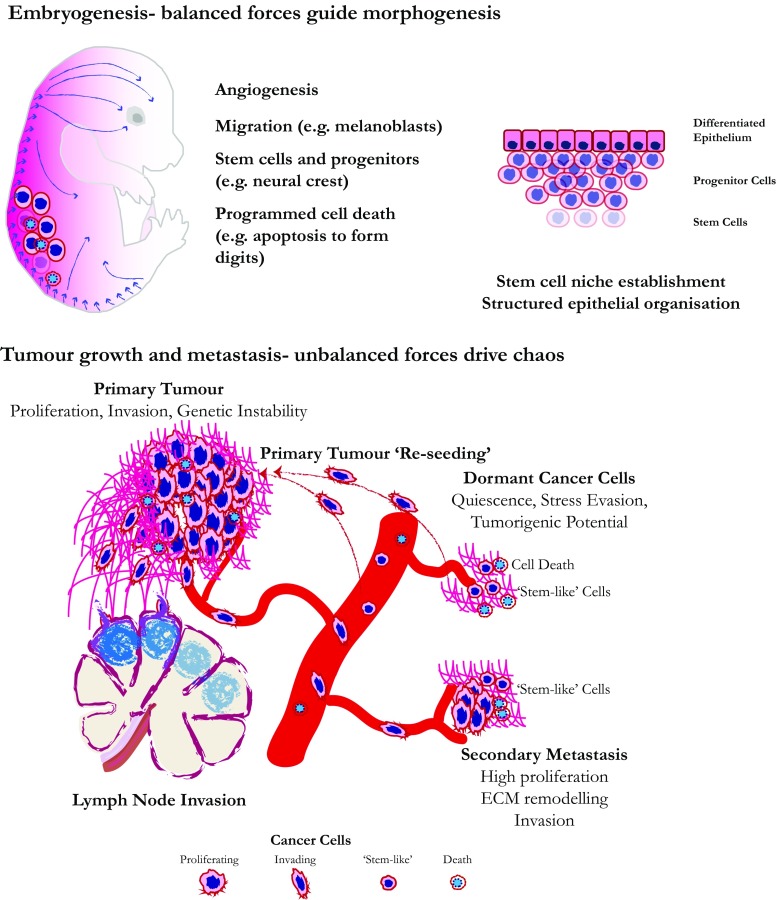
During embryogenesis, forces balance as cells proliferate, differentiate and sort into specific tissues and organs. Angiogenesis allows oxygenation of the growing embryo and migration, both collectively and individually, drives sorting and homing of cells and tissues. Embryonic tissue shows plasticity in cell fate, but as development progresses, cells become more committed, and stem cells form in specific niches, where they continue to maintain tissues and organism in the adult. Programmed cell death is also important for pruning out cells during sculpting, such as in the formation of digits. The differentiated epithelium (shown right) is an example of a tissue that maintains stem cells in a niche, progenitor cells and differentiated cells in a continuous state of equilibrium in the adult. There is much less cell motility in adult tissues than embryonic, and growth is generally balanced by death and pruning. Unlike the well-organised embryo, tumours behave in more unpredictable and chaotic ways. However, in common with embryos, they show increased angiogenesis and cell migration. The blood vessels in tumours are generally leaky and tortuous, resulting from and causing further force imbalances. Tumours also have stem-like cells and have altered capacity for proliferation, often hyperproliferating or suppressing programmed cell death to become crowded and deprived of nutrients. If the stem-like cells escape from the primary tumour, they may land in lymph nodes or travel through the bloodstream, where they can seed new tumours (metastases) at distant sites. Most escaping tumour cells are thought to die due to the hostile conditions and the body’s surveillance system, but if even a few survive, they can start new tumours. New tumour formation can start immediately or after years of dormancy, a poorly understood state where the cells lie in the host tissue, but the tumour is not detectable. Dormancy may be quiescence and fails to grow, or may be a balance of growth and death that keeps the small cluster undetectable. However, these small micrometastases re-awaken and can result in full metastasis. Metastases can also shed cells into the bloodstream that return to the primary tumour and increase its aggressiveness and diversity

While normal tissue development follows an orderly programme, cancer and metastasis are chaotic. During development, stem cells give rise to more differentiated precursors and migration follows orderly programmes. Blood vessels invade tissues and form networks to deliver oxygen and nutrients (Fig. 1). ECM mechanics guide developmental migration, stem cell formation and organogenesis (reviewed in (Kumar et al. 2017)). Physical forces in normal tissues are balanced to maintain identity and architecture (Butler and Wallingford 2017; Gilbert and Weaver 2017; Vijayraghavan and Davidson 2017). During tumorigenesis, aspects of the developmental process can be mimicked, but in a chaotic way (Fig. 1). The balance that maintains normal tissue architecture is lost by overgrowth and inappropriate matrix deposition, leading to increased cell crowding and nutrient starvation. These changes promote migration away from the primary tumour into the extracellular matrix or invasion into the lymph or vascular systems. Cancer cells can also be shed into the imperfect tumour vasculature and gain access to the circulation to disseminate widely. The vast majority of disseminated tumour cells die, either from shear forces in the blood or because they land in a hostile environment. However, if even one cell in a million survives, it can gain the potential to form a new tumour or to lie dormant in a tissue until conditions trigger new tumour formation. Disseminated cells can land in a niche that promotes stem cell characteristics or alternatively make their way back and colonise in the primary tumour and thus increase its heterogeneity and aggressiveness (Kim et al. 2009) (Fig. 1).

This review will focus on how mechanical constraints or imbalances, imposed by the extracellular matrix and cell crowding of malignant tissues, shape cell behaviour and drive tumour progression and metastasis. We will also discuss how biophysical methods and engineered environments could provide reliable in vitro platforms to measure mechanical force imbalances and determine their consequences for cancer cell behaviour. We highlight the need for a comprehensive biophysical approach to better understand the interactions between the cancer cells and their environment, ultimately facilitating the design of novel and effective therapeutic strategies.

## ECM mechanical properties—I. Rigidity sensing governs proliferation, migration and identity

**Fig. 2:**
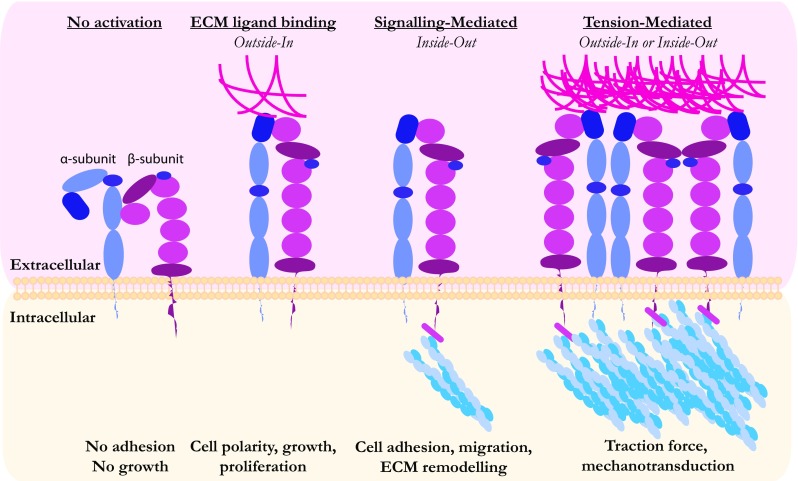
Integrin activation and importance for balanced growth. Integrins lie at the roots of cellular mechanosensing, as they are considered to be the main membrane receptors mediating cell-ECM interactions. They are heterodimers of α- and β-subunits forming an elongated extracellular ligand binding domain and a short cytoplasmic tail. In the absence of stimuli, integrin subunits have an inactive bent conformation. Integrin subunit elongation and activation can occur either through ECM protein ligand binding on the extracellular site (‘outside-in’) or by intracellular signalling events mediated mainly by focal complex or actin cytoskeleton associated protein such as talin (‘inside-out’). Integrin activity can enhance remodelling of the surrounding microenvironment which can also promote more integrin activation indicating a positive loop. Tension and mechanical force arising either from ECM or cytoskeletal dynamics can also extend, activate and cluster integrin subunits. Non-transformed cells require a degree of ECM adhesion and integrin signalling to sustain their proliferation and growth. Malignant transformation, however, maintains cell proliferation even in the absence of ECM adhesion. At the same time though, transformed cells display integrin enrichment and imbalanced cell-ECM dynamics. Tumours frequently display an increase in ECM stiffness, which can be further enhanced by inflammation and fibrosis. This can drive increased cytoskeletal activation as well as signalling downstream of integrin activation

Normal cells display anchorage-dependence, a process by which cells sense adhesion to the ECM via transmembrane receptors, especially integrins, which signal to the nucleus to regulate proliferation and survival. Integrins bind to ECM ligands, such as fibronectin or collagen, mainly through their arginyl-glycyl-aspartic acid (RGD) motifs. Binding and tension against the substratum cause integrins to undergo a conformational change promoting their activation and clustering, triggering adhesion and proliferation (Schwartz 2010). The controlled presentation of ECM molecules on normal epithelial tissues can maintain and regulate the homeostasis of tissue growth and architecture. However, during tumorigenesis, extensive ECM remodelling and deposition of a different repertoire of ECM molecules by cancer cells and cancer-associated fibroblasts perturb this balance. Furthermore, genetic changes in the tumour cells, frequently leading to increased Ras and MAP kinase signalling, render them anchorage-independent (Kang and Krauss 1996). Strikingly, Ras GTPases can also activate integrin-dependent signalling cascades in an adhesion independent manner, a process known as ‘inside-out’ integrin activation (reviewed in (Kinbara et al. 2003), see Fig. 2). All of these changes impact on control of proliferation and survival, allowing cancer cells to override signals from a hostile environment designed to eliminate them.

**Fig. 3:**
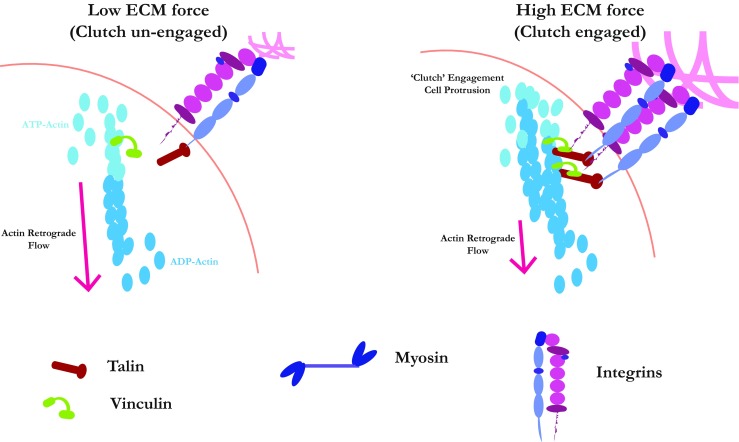
Cells generate force against stiff ECM, leading to clutch engagement. When cells experience soft or viscous matrix, where adhesions do not generate enough tension to stretch mechanosensitive proteins and trigger a response, the molecular clutch remains unengaged. In this situation, actin polymerisation at the leading edge is uncoupled from adhesion, and retrograde flow of newly generated filaments occurs in the direction away from the plasma membrane. Adhesions remain small, and the cell is not able to use actin-based protrusion to move against the substratum. However, upon a threshold of ECM stiffness, mechanosensitive cytoskeletal linkers, such as vinculin and talin, engage and form a molecular clutch. The clutch catches the ECM-derived force and transmits it to the cytoskeletal cortex. As adhesions increase in size due to integrin clustering and the cytoskeleton couples to the rigid matrix, actin polymerisation results in membrane protrusion and promotes motility. During tumorigenesis, high ECM stiffness, enrichment and hyperactivation, the mechanosensing machinery can promote invasion, migration and metastatic dissemination

Integrin adhesions not only control proliferation but also motility, via direct connections with the actin cytoskeleton. Vinculin and talin are mechanosensitive proteins that couple actin to integrins at focal adhesions. They form what is termed a molecular clutch (Fig. 3) whereby actin polymerises and is pushed and pulled back from the plasma membrane toward the cell centre by myosin-II in a phenomenon known as retrograde flow. When the clutch is engaged on a rigid substratum, the actin tethers to the focal adhesions and force is generated to drive motility. When cells are on a softer substratum, the clutch is less engaged and adhesions are weaker—preventing accumulation of the tension that drives forward translocation of the cell. In particular, talin can be periodically stretched in an actin flow dependent manner, revealing cryptic vinculin-binding sites on the talin molecule (del Rio et al. 2009; Wang 2007). Rigidity sensing is mediated by a series of cytoskeletal-dependent contraction forces on the edge of the cells (Iskratsch et al. 2014). Essentially, it seems that cells sense their underneath matrix by contracting it through a series of sequential events involving actin polymerisation and focal complex assembly and reinforcement. How cells sense and respond to stiffness is still a very active area of study, and a recent screen for receptor tyrosine kinases (RTKs) involvement implicated Axl and ROR2 phosphorylating tropomyosin 2.1, myosin IIA and filamin A (Prager-Khoutorsky et al. 2011; Yang et al. 2016). These signalling pathways provide a direct connection between the cytoskeleton and RTKs in mechanosensing, which could have broad implications for cancer if they turn out to be general.

Do cancer cells sense rigidity? Or have they lost this control? A recent study demonstrates that some cancer cells can maintain high proliferation rates even on low adhesion environments (Yang et al. 2018). However, physical and chemical gradients in the tumour microenvironment are crucial for tumour progression (Oudin and Weaver 2016) suggesting that transformed cells still sense and respond to ECM rigidity. Tumours not only secrete more matrix than normal tissues, but they remodel it differently, leading to increased stiffness, breach of basement membrane barriers and hypoxia. Different types of collagens, fibronectin, tenascins and other ECM molecules are abundant in the microenvironment of tumours (for examples, see Box 1); these contribute to the alteration of ECM mechanical properties. In addition, tumour ECM becomes infiltrated by immune cells and fibroblasts, which deposit ECM as well as increasing crowding, pressure and nutrient consumption. Both tumour cells and surrounding cancer-associated fibroblasts (CAFs) show enhanced expression of the collagen crosslinking catalyst lysyl oxidase (LOX) (Erler et al. 2006; Erler and Giaccia 2006; Miller et al. 2015). Collagen crosslinking increases ECM stiffness and promotes invasion and cancer malignancy (Levental et al. 2009). LOX expression may also increase in the pre-metastatic niche leading to changes that promote survival or growth of metastases (Erler et al. 2009). Furthermore, it has been suggested that Caveolin-1 expression by CAFs increases ECM stiffness in the tumour microenvironment promoting cell invasion. Caveolin-1 can control the phosphorylation of the RhoGAP p190, an important regulator of Rho GTPase activity. This results in defective contractility and increased invasiveness of fibroblasts but also to the deposition of an altered highly crosslinked collagen matrix (Goetz et al. 2011). Together, these increase tumour ECM stiffness, a property that is currently emerging as one of the most important biophysical manifestations of the tumour microenvironment.

ECM stiffness promotes matrix remodelling and invasion via signalling pathways such as FAK-dependent activation of Rac1 (Bae et al. 2014; Charras and Sahai 2014). Cancer cells respond to increased stiffness by assembling invadopodia, actin-rich structures that not only exert force on the matrix but also engage matrix metalloproteases, which degrade ECM (Eddy et al. 2017; Haage and Schneider 2014; Yu et al. 2012). Invadopodia and actin-based protrusions are important mediators of invasion and metastatic spread of pancreatic tumours in vivo (Li et al. 2014). In pancreatic tumours with SMAD4 mutations, ECM stiffness was linked to high STAT3 signalling activity inducing increased tension and fibrosis, favouring an aggressive phenotype (Laklai et al. 2016). Furthermore, ECM stiffness can promote angiogenesis, altering normal vasculature integrity to mimic cancer-associated vasculature (Bordeleau et al. 2017).

ECM stiffness impacts on gene expression signatures in both normal and tumour tissues, enhancing programmes that determine cell identity and differentiation or stemness. ECM stiffness is linked to induction of epithelial-to-mesenchymal transition (EMT), a developmental process that goes awry in cancer and is linked to progression of epithelial cancers such as PDAC (Krebs et al. 2017; Morris and Machesky 2015). Specifically, Twist1 is a critical transcriptional regulator that acts as an EMT promoter and is regulated by increased ECM stiffness, favouring invasion and metastasis (Wei et al. 2015). In addition to integrin-actin connections, the nucleus is coupled with adhesions and actin to cause transcriptional changes that regulate many tumour-promoting processes. The nuclear translocation of two transcriptional co-factors, Yap and Taz, mediates transcriptional responses to ECM mechanosensing in many cells and tissues (Panciera et al. 2017). Yap/Taz and the transcriptional factor TEAD are part of the well-known Hippo pathway, an evolutionarily conserved developmental pathway that controls tissue morphogenesis and homeostasis (Panciera et al. 2017).

ECM rigidity triggers integrin clutch engagement and leads to nuclear translocation and activation of YAP/Taz signalling (Halder et al. 2012). In the absence of mechanical stress, Yap/Taz are localised to the cytoplasm where they can be phosphorylated by LATS1 and turned over in the proteasome (Panciera et al. 2017). Activation of Yap/Taz signalling triggers a transcriptional programme that affects cell stemness and differentiation (Lian et al. 2010). Multiple targets downstream of Yap/Taz are affected by mechanosensing, including the matricellular matrix protein CCN1, which promotes cancer cell intravasation and metastasis (Reid et al. 2017). Another transcriptional regulator FHL2 (four-and-a-half LIM domain family protein 2) translocates to the nucleus on soft substrates, where it induces the transcription of p21, negatively regulating cell proliferation (Nakazawa et al. 2016). While mechanosensitive transcriptional targets are beginning to be uncovered, much more needs to be done to fully understand how mechanosensing impacts on cell identity and differentiation. Furthermore, although some actin regulators have been implicated in Yap/Taz connection to the cytoskeleton (Aragona et al. 2013), the connections between Yap/Taz and the molecular clutch warrant further investigation.

**Fig. 4:**
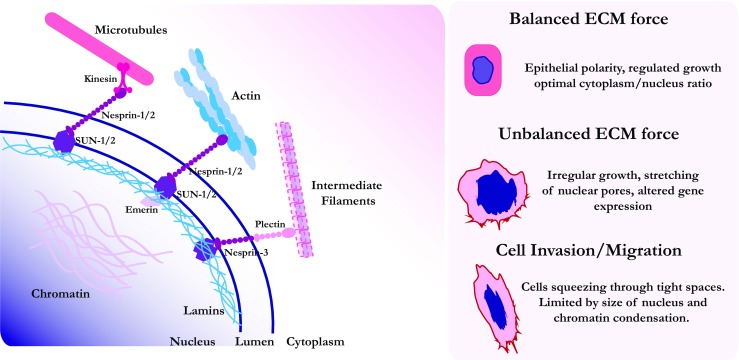
Nuclear forces are balanced by the cytoskeleton. The nucleus is connected to the cytoskeleton via transmembrane proteins, including nesprins and SUN proteins. These assemblies are called the LINC, linker of the nucleoskeleton and cytoskeleton complex. The LINC complex connects to the cytoskeleton, including actin filaments, microtubules and intermediate filaments through the nuclear envelope to chromatin. The LINC complex is usually composed by the SUN protein subunits connected to lamins intranuclearly and the nesprin proteins on the cytoplasm. This complex is thought to relay cytoskeletal changes to alterations in chromatin organisation and affect gene expression. Additionally, increased force can lead to stretching of nuclear pores and increased exchange of proteins between the nucleus and the cytoplasm. When cells invade through pores of the ECM or intravasate into a blood vessel and travel through the bloodstream; the associated squeezing and shear forces affect chromatin organisation and stability of the genome

The nucleus is physically connected with the cytoplasm and is under stress in normal cells. Disruption of this connection affects its size and shape with important implications for genome function (Mazumder and Shivashankar 2010). In particular, the nucleus is coupled to the actin cytoskeleton and focal complexes via nesprins and the nuclear LINC complex (Lombardi et al. 2011). The LINC complex consists of nesprins, KASH and SUN proteins that span the nuclear membrane and interact both with chromatin and the actin cytoskeleton (Rothballer et al. 2013) **(**Fig. 4**)**. The LINC complex regulates cell cycle progression in response to stress, for example in *Drosophila melanogaster* muscle (Wang et al. 2018a)*.* In addition to transmitting force to chromatin, ECM stiffness couples with nuclear pores, exposing their interiors to the cytoplasm and thus triggering active nuclear import. This is thought to work by causing captured protein targets, including YAP, to unfold and be imported from the cytoplasm (Elosegui-Artola et al. 2017). Mechanisms of this increased import are still unknown, but perhaps nuclear softening, due to altered expression of lamins, could further enhance mechanosensitivity.

One of the most direct ways that cell mechanosense is via ion channels. Ion channels are pore-forming transmembrane proteins that control the flow of ions across the cell membrane. They can be rapidly influenced by ECM derived force or pressure, regulating a variety of cell behaviours. Specifically, the Piezo channel is a massive 38-transmembrane spanning channel that translates mechanical stimuli into calcium signals (Wang et al. 2018b; Zhao et al. 2018). Piezo is important for stem cell mechanosensing in the *Drosophila* midgut, mediating proliferation and differentiation (He et al. 2018), as well as for touch sensation in neurons (Ranade et al. 2014; Woo et al. 2015). When cells crawl through a confined space, Piezo is activated to increase intracellular calcium levels, leading to negative regulation of protein kinase-A (Hung et al. 2016). This pathway works in concert with myosin-II to sense confinement and regulate cell migration, as well as setting up a positive feedback of myosin-II-activated calcium influx (Hung et al. 2016). Piezo is implicated in pressure-induced pancreatitis, a form of pancreatic inflammation resulting from trauma, duct obstruction or any medical procedure that puts pressure on the pancreas (Romac et al. 2018). Inhibiting Piezo can reduce pancreatitis, suggesting potential for therapy and perhaps scope for further exploring a role of Piezo channels in pancreatic cancer. Considering also the deregulated calcium signalling that cancer cells exhibit and that targeting calcium signalling emerges as a potential cancer therapy (Cui et al. 2017), elucidating how ECM stiffness is communicated within the cancer cells by ion channels will be crucial to understand promotion and dissemination of malignancy.

Among their multiple functions, Rho-family GTPases emerge as major signal transducers of ECM stiffness sensation. In particular, RhoA is one of the most important actomyosin regulators, and Rac1 mediates new actin assembly stimulating a plethora of downstream events. Piezo activation causes RhoA activation in response to mechanosensing in cancer cells (Pardo-Pastor et al. 2018). In addition, the Rho-GEF obscurin mediates RhoA activation in breast cancer in response to increased ECM stiffness (Stroka et al. 2017). STEF/TIAM2 RacGEF mediates Rac activity in concert with NMMIIB to maintain the cell’s perinuclear actin cap (Woroniuk et al. 2018). The perinuclear actin cap is an actinomyosin structure connecting the nucleus to the actin cytoskeleton via nesprin and SUN proteins (Chambliss et al. 2013). Mechanical stimulus triggers the actin cap to relay signals to the Yap/Taz pathway as well as maintaining nuclear structure and orientation during migration (Diamantopoulou et al. 2017). Considering the multiparametric role of GTPases in cancer progression, it will be worth investigating how the aforementioned pathways are affected by ECM-derived force in tumorigenesis.

### Modelling stiffness in vitro

**Fig. 5:**
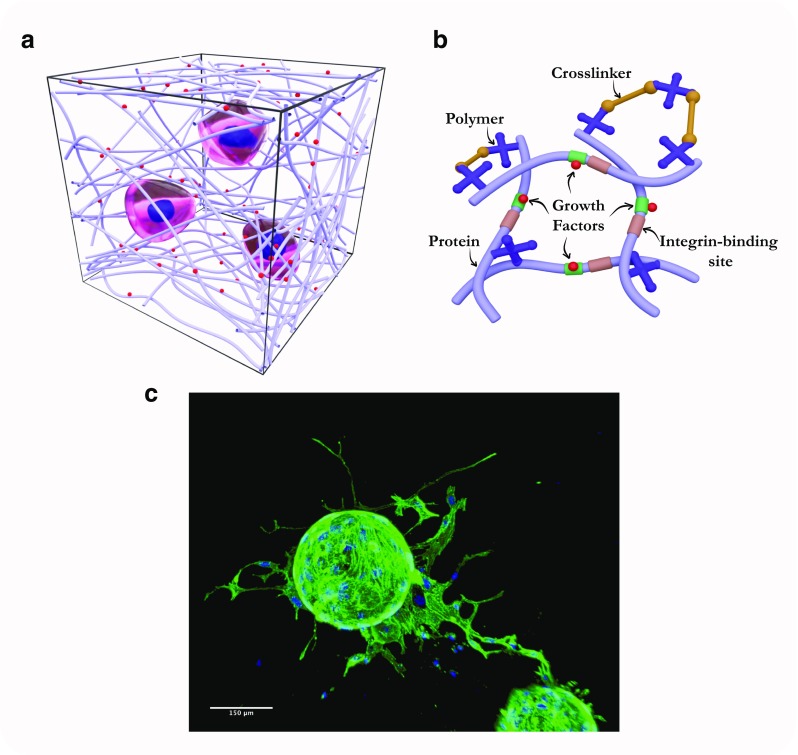
Hydrogels recapitulate mechanical aspects of the microenvironment. **a** Sketch of a hydrogel, showing cells embedded in the 3D environment. **b** Details of an example hydrogel, showing crosslinker, which can be varied to control pore size and stiffness; polymer, which can also be varied to change mechanical and chemical properties; protein, which can represent an endogenous tissue or tumour matrix protein such as fibronectin; growth factor, which can be included in the hydrogel and presented either upon stimulus or constitutively. **c** Micrograph showing spheroid of mouse pancreatic cancer cells growing in a hydrogel. Sketches courtesy of Sara Trujillo-Munoz, University of Glasgow

The first and still most commonly used materials to recapitulate the ECM of tumours in vitro are natural ECM-derived components, including fibronectin, collagen, cell-derived matrices or reconstituted basement membranes. Their major advantages over artificially generated systems are their intrinsic biocompatibility and cell adhesion properties. However, there is a need to engineer surfaces that not only mimic biomechanical properties of the ECM but also offer the option to control dynamics, degradability and protein composition, while maintaining other properties. Standard 2D systems for probing the mechanoresponsiveness of cells have included either PDMS (polydimethylsiloxane) surfaces or hydrogels usually composed by acrylamide. The latter can be mechanically tuned by varying the crosslinker concentration to modulate the stiffness and incorporate RGD adhesive peptides to facilitate cell adhesion (Kandow et al. 2007). Alginate (a polysaccharide derived from algae) and reconstituted basement membrane are also materials that can be incorporated into a synthetic interpenetrating polymer network. Their stiffness can be modulated by altering the ionic crosslinking of alginate, without changing other parameters including polymer concentration (Chaudhuri et al. 2014). Recent innovations allow the production of controllable synthetic hydrogels that support organoid and cancer spheroid growth. These offer exciting opportunities for studying cell behaviour in 3D allowing complex cellular co-cultures and defined physical properties (Cruz-Acuna and Garcia 2017). Polyethylene glycol (PEG) and poly(lactide-co-glycolide) (PLG) are commonly used to control mechanical properties in 3D hydrogels. They are often engineered to incorporate cell adhesion ligands as well as biodegradable crosslinkers to increase bio- and cyto-compatibility. The stiffness of those synthetic 3D hydrogels can be varied by changing the length and density of crosslinkers and have already been applied to studies of cancer cell properties, including growth, invasion and migration (Singh et al. 2015) (Fig. 5). Not only stiffness but also composition is important. It is worth noting that complex 3D systems require precise characterisation to identify the exact properties that the encapsulated cells sense. In addition, cells interact dynamically with their milieu, an interaction that includes degradation, secretion and deposition of extracellular molecules (Ferreira et al. 2018).

Since ECM stiffness changes dynamically through extensive remodelling and protein deposition, it is important to generate smarter materials that will allow us to study how cells respond to dynamic, periodic or reversible alterations of the mechanical properties. Classic synthetic hydrogels are irreversibly remodelled by cells, and their mechanical properties usually cannot be tuned after their generation. However, recent chemical developments allow novel material applications to engineer 3D microenvironments that can be rapidly and reversibly modified in a controllable manner—reviewed in (Rosales and Anseth 2016). For example, the use of photoswitchable crosslinkers has allowed stiffening of a synthetic hydrogel upon light stimulus (Frey and Wang 2009; Guvendiren and Burdick 2012; Lee et al. 2018; Yeh et al. 2017). Enzymic reactions have also been recently applied to mediate stiffening of hydrogels in situ (Liu et al. 2017). Thus, it is now possible to dynamically assess cancer cell responses to acute and local changes of the stiffness of their environment in controlled conditions. This could be especially relevant in the pre-metastatic niche, where immune cell activity, wounding or trauma might trigger awakening of dormant cancer cells and promote metastatic growth.

## ECM mechanical properties—II. Viscosity has a similar role to rigidity, but is relatively unexplored

### Viscoelasticity of the tumour matrix is an understudied relative of stiffness

While stiffness is an established driver of biological mechanosensing, the importance of viscosity and viscoelasticity of the ECM is just beginning to be understood. Elastography on human patients showed malignant breast tumours to be more viscous or fluid-like than benign lesions, suggesting physiological relevance (Sinkus et al. 2007). In addition, interstitial fluid in the tumour niche might contribute to the viscous properties of the ECM. Viscosity engages the molecular clutch in much the same way as stiffness does, and triggers adhesion assembly and Yap/Taz signalling (Bennett et al. 2018). Changing matrix composition, including differential expression of collagens, laminins and fibronectin, as well as accumulation of hyaluronan and other viscous ECM components will create an altered viscosity in the tumour microenvironment. Hyaluronan accumulation, for example, correlates with increased cancer stemness and aggressiveness of tumours (Chanmee et al. 2016a, b). Further research is needed to unravel the contribution of those viscous properties on tumour progression. Matrix viscoelasticity impacts proliferation and cell spreading by mechanisms that are not yet understood (Bauer et al. 2017; Chaudhuri et al. 2015). Identifying the liquid/solid states of desmoplastic tumours, such as pancreatic ductal adenocarcinoma, could open up new therapeutic possibilities.

Not only ECM viscosity, but viscosity of the tissue at the level of cell-cell interactions, governs normal and cancer cell organisation. Tumours contain masses of tightly packed cells, which have been described as physically jammed. It is mechanically challenging for packed cells forming cell-cell junctions to flow past each other or move freely. This has been modelled in vitro using cell monolayers, which are fluid during low confluency, but then jammed as the cells proliferate continuously and pack more tightly in a confined space (Chepizhko et al. 2018). Upregulation of endocytic trafficking can un-jam epithelial cancer cells, promoting flow and collective movement. In particular, the small GTPase Rab5a, an important mediator of endocytosis, induces collective cell motility upon physical constraints and jammed monolayers, a process that is interrupted by increasing fluid efflux (Malinverno et al. 2017). In addition, E-cadherin trafficking is thought to play a major role in unjamming cells (Song et al. 2013). Many tumours still express E-cadherin, and its mobility correlates with metastatic potential (Erami et al. 2016). Cancer predominantly invades in a collective manner, and thus it will be important to study the viscosity of invasion streams and surrounding matrix to inform about likelihood of metastasis or response to treatments.

### Modelling viscoelasticity in vitro

Various models are in development to model ECM viscoelasticity. Engineered lipid bilayers can be manipulated to present different cellular stress relaxation properties (Bennett et al. 2018). Interestingly, it is also possible to generate hydrogels of constant stiffness but of variable viscoelasticity. This is achieved by modifying the molecular weight of the crosslinkers and therefore their mobility (reviewed in (Chaudhuri 2017)). It would thus be possible to test whether dynamic-mechanical phenomena (e.g. stress relaxation) could trigger awakening of dormant cancer cells or affect invasive capabilities. This new idea bears testing, as tissues and tumours are differently viscoelastic in nature, and their properties change over relevant timescales. For example, when the lungs inflate and deflate, shear stress is created and even small changes in viscosity may lead to increased epithelial damage (Chen et al. 2015). This damage could activate an increased stretch response in dormant cells, as well as causing local inflammation and thus affecting recurrence of lung cancer or lung metastases of other cancers.

A key study from Shenoy and colleagues highlights the most relevant parameters to consider for modelling the importance of viscosity on cell spreading. These are the timescales for binding of the molecular clutch, the lifetime of engagement of the molecular motors and the substrate relaxation times (Gong et al. 2018). Only by comparing the timescales of cellular events with substrate relaxation events can we reveal the impact of viscoelastic properties on cell behaviour. These authors concluded that for soft substrates, there was an optimal viscosity with characteristic relaxation time that slows down the response to cell pulling and stiffens the material and thus promotes cell spreading. In contrast, on rigid substrates, viscosity made little difference to cell engagement since the bound clutches are already saturated by stiffness. Importantly, this study used three different types of hydrogels to demonstrate these effects, including hyaluronic acid, alginate and polyacrylamide, with biological matrix molecules such as collagen incorporated. They also used different cell types to show robustness at the biological level and supported their conclusions with a Monte-Carlo model. Another recent study used encapsulation of deformable high molecular weight long linear polyacrylamide within crosslinked polyacrylamide hydrogels to have independent control of elasticity and viscosity and model soft tissues (Charrier et al. 2018). Use of these new materials revealed that differentiation of hepatic stellate cells could be dependent on viscosity, showing a relevance of viscosity in biological processes. Further development of tuneable viscosity hydrogels will enable a thorough study of viscosity.

Tightly packed cells such as in epithelial monolayers have been compared with particles in a tightly packed suspension, which can jam when the temperature is low, the density is high and the suspension acquires a yield stress. Cell-cell viscosity in jammed epithelia has been mathematically modelled, and although this is still a relatively new idea, studying the jamming transitions using models developed for physical systems may be applicable to biological systems (Gamboa Castro et al. 2016). Cells of mesenchymal or epithelial phenotype were mixed together in varying densities. Velocity was measured as a function of density, which revealed that motility arrest occurred in certain conditions and could be modelled similar to jamming in physical systems. However, another study of cell jamming argues that cellular contraction and adhesion are key components of motility behaviour that are overlooked in such models, challenging therefore the idea that cells behave like particles in a suspension (Vig et al. 2017). More studies are needed to determine the usefulness of the various analogies and models.

Recent developments in tissue decellularization techniques allowed the isolation of various native ECM environments from whole organs and subsequent study of viscoelastic properties. In particular, in situ decellularization of tissues (ISDoT) not only allows decellularization of whole organs but also seems to leave ECM architecture intact (Mayorca-Guiliani et al. 2017). That allowed proteomic mapping of the ECM components and could also facilitate correlation of such profiles with viscoelastic mechanics of different ECM environments including for example pre-metastatic and metastatic niches. Study of decellularized tissues could also promote the design of more intricate ECM-mimicking materials. Such advances could reveal the contributions of ECM viscoelastic properties to tumour progression.

## ECM architecture—I. Density, linearity and alignment govern migration and cell identity

### Matrix fibre alignment reinforces migration patterns and enhances stiffness signals

In addition to stiffness, tumour matrix displays abnormal architecture: typically, fibres align radially away from the centre of tumours and are frequently bundled into highways traversed by cells at the invasive edges (Han et al. 2016; Sander 2014). Fibre alignment promotes invasive behaviour and has been modelled using collagen gels (Ahmadzadeh et al. 2017; Fraley et al. 2015). Additionally, collagen alignment has been correlated to alpha-SMA expression indicating a transformation of normal residing pancreatic fibroblasts, known as stellate cells, toward cancer-associated fibroblasts (Drifka et al. 2016). Thus, both tumour and stromal cells are transformed to a more aggressive phenotype by fibre alignment. Fibre alignment not only affects migration but also may contribute to hypoxia at the centre of tumours, setting up a self-reinforcing pro-metastatic programme. High-density collagen hydrogels triggered cancer cells to migrate and degrade their surrounding matrix when they were under hypoxic conditions (Lewis et al. 2017). Hypoxia promotes changes in composition and remodelling of the ECM. The hypoxia-inducible factor 1 (HIF-1) alters ECM deposition and remodelling genes to promote fibre alignment, stiffening and further intensifying hypoxia (Gilkes et al. 2013). A correlation between collagen architecture and hypoxic areas has also been reported in vivo (Kakkad et al. 2010). Interestingly, alignment of collagen fibres is correlated with reduced survival in a cohort of 114 PDAC patients (Drifka et al. 2016). It is worth exploring whether fibre alignment additionally might set up barriers to chemotherapy and immune therapy and exploring how immune cells react to the radially aligned tumour matrix.

### Modelling fibre alignment in vitro

Collagen is one of the most commonly used biopolymers to study 3D cell behaviour in vitro*.* The study of collagen architecture has been facilitated by the development of advanced optical techniques, including second-harmonic generation (SHG) microscopy (Vennin et al. 2018) which takes advantage of the helical arrangement of collagen (see Box 1) to image scattered photons. SHG imaging of human tumours, combined with other stromal markers, associated collagen ECM architecture with PDAC progression (Drifka et al. 2016). Another promising method, liquid crystal–based polarised light imaging, provides label-free imaging of collagen fibre orientation and alignment (Keikhosravi et al. 2017). Collagen matrix alignment can be performed, and cell migration was studied in vitro using methods such as rotational 3D alignment of collagen fibres (Nuhn et al. 2018) and reviewed in (Wolf et al. 2009). Self-assembling 3D collagen matrices engineered with the crosslinking enzyme transglutaminase II have been informative of the role of matrix alignment and topography to MMP activity in cell migration (Fraley et al. 2015). The stiffness of 3D collagen gels can be controlled using glycation, a monosaccharide-dependent modification of collagen residues. This modification can increase the rigidity of the gels without affecting architecture (Bordeleau et al. 2017; Nuhn et al. 2018). Fibre alignment can be accompanied by another change, with impact on bone metastasis and mineralisation. The latter is essentially a composition of type I collagen fibrils with intrafibrillar crystals of non-stoichiometric carbonated hydroxyapatite. A polymer-induced liquid-precursor (PILP) process has been applied to mimic intrafibrillar collagen mineralisation in vitro, demonstrating that collagen mineralisation can increase cell motility (Choi et al. 2018). Some PDAC tumours show mineralisation, but this has not, to our knowledge, been correlated with fibre arrangement or invasiveness and may be interesting for future study.

While reconstitution of collagen matrix provides important insights, synthetic fibres offer increased control of mechanical properties and alignment on nano-, meso- and micro-scales. Electrospinning, a method whereby an electrical field is used to draw viscoelastic polymer solutions out of a reservoir and by electrical repulsion, causes them to jet into a thin filament that is a longstanding technique to generate fibres of controlled composition, alignment and physical properties (Pham et al. 2006). Recently, electrospun fibres have been combined with native ECM proteins, such as laminin and collagen (Kwon et al. 2017), to reconstitute 3-dimensional scaffolds for cells and tissues. By manipulating the alignment of electrospun fibres, it is possible to recapitulate in vivo architecture, such as those found in wounds (radial) or tendons (uniaxial) (Pham et al. 2006). An alternative to electrospinning is flow spinning, where a fluid reservoir draws out the jets of viscoelastic polymers into fibres with various dimensions and topology (Madurga et al. 2017). The fibres are aligned onto substrates of desired dimension in the centre of the well. This method avoids high voltages and may be more biocompatible.

## ECM architecture—II. Geometry: Confinement and topography

### Matrix geometry influences migration and tumour progression

Curvature is another important consideration of ECM, as cells contain curvature sensing proteins, and, for example, nanopit-patterned surfaces decrease cell adhesion compared to flat substrates (Martines et al. 2004). The BAR domain comprises a curved protein domain that self-assembles and can sense curvature or induce curvature in membrane surfaces (Chen et al. 2012). Bar proteins interact with small GTPases, such as Rac1, and can influence signalling, cytoskeletal architecture and membrane dynamics (reviewed in (Vogel and Sheetz 2006)). BAR domain–containing proteins also generate curvature on endocytic membranes, and they can possibly drive formation of filopodia and lamellipodia, structures that trigger cell motility and dissemination of cancer cells (Heath and Insall 2008). Some BAR proteins are upregulated or mutated in cancer and have been implicated in EMT (Chen et al. 2012). BAR proteins also contribute to the invasiveness of cancer cells, promoting invadopodia formation (Pichot et al. 2010; Yamamoto et al. 2011). In addition to BAR proteins, the nuclear LINC complex is implicated in curvature sensing, via transmission of stretch when a cell is on a convex surface. When tested on cell-sized nano-pits, cells positioned themselves into concave pits where the nucleus was under the least tension (Pieuchot et al. 2018; Werner et al. 2017). It is intriguing to ask whether membrane curvature alterations might also contribute to the reawakening of quiescent or dormant tumour cells.

Related to curvature is pore size, another important property of ECM that varies widely in vivo and in cancer. ECM porosity in particular has been studied extensively in relation to cell migration (reviewed in (Charras and Sahai 2014)). Development of a 3D cell culture system uncoupling collagen concentration from collagen gel microarchitecture indicated that cancer cells acquired a more motile and invasive phenotype when exposed to small pores (Carey et al. 2012). Migration through small pores has been linked to DNA damage and genomic instability (reviewed in (Isermann and Lammerding 2017)). A migrating cell can squeeze through very small openings, sometimes down to a few microns in diameter, but is limited by how much it can compact its nucleus. Extreme nuclear compaction can damage the nuclear envelope inducing increased exchange between cytoplasmic and nuclear proteins (Denais et al. 2016). To overcome limited ECM pore size, cancer cells can employ proteolytic activity and ECM degradation (Wolf et al. 2013). Not only are pores limiting, but nuclear squeezing during migration can lead to rupture and increased genomic instability. For example, cytoplasmic nucleases could enter into the nucleus causing DNA damage (Irianto et al. 2017). In addition, normal cells have mechanisms to repair nuclear envelope rupture (Olmos et al. 2015, 2016; Vietri et al. 2015), such as the endosomal sorting complexes required for transport (ESCRT) machinery (Isermann and Lammerding 2017). Defects in the repair of nuclear envelope ruptures during migration through restricted ECM pores could further contribute to cancer aggressiveness. Since the nucleus is mechanically coupled to the actin cytoskeleton (Fig. 4), it is also vulnerable to the forces transmitted through it. There is evidence that ECM stiffness increases genome instability (Pfeifer et al. 2017). DNA damage caused by migration through constricted pores can hinder the proliferation of cancer cell lines (Pfeifer et al. 2018). It is not yet clear how significant the effect of matrix geometry is on DNA damage in vivo, as other factors (e.g. DNA repair mechanisms) also play a major role.

Interestingly, ECM geometry and confinement can also regulate signalling pathways, including YAP signalling. Cell confinement and spreading can induce Yap nuclear translocation (Dupont et al. 2011), as can stretching or inducing curvature to a confluent monolayer (Aragona et al. 2013), with mechanical stress being transmitted through cell-cell junctions (Benham-Pyle et al. 2015). Thus, it seems that the curvature and the topography of the ECM could be important regulators of YAP activity in cancer. Apart from the confinement of cancer cells or proteins, ECM nano- and micro-conformation could also confine diffusible factors in limited spaces. These could signal to cancer cells and trigger chemotactic responses with important implications to cancer spread (Tweedy et al. 2016).

### Modelling ECM topography in vitro

When trying to model ECM topography, an important challenge is how to uncouple it from intrinsic mechanical properties, such as viscoelasticity. Carey et al. recently presented an improved collagen gel culture system, where collagen porosity could be studied independently from concentration (Carey et al. 2012). In addition, semi-3D microfabricated substrates have been applied to mimic confined microenvironments (Booth-Gauthier et al. 2013). 3D microchannel scaffolds of collagen and glycosaminoglycan have been used to model ECM porosity to study fibroblast migration (Harley et al. 2008). Microfluidic devices are useful to study cell migration in conditions that could mimic cell crawling inside the tissues in vitro (Irimia et al. 2007). Recent advances include the incorporation of native decellularized tissue ECM into tissue matrix scaffolds to fabricate porous hydrogel systems with tissue-like architectural integrity (Rijal and Li 2017). Synthetic porous hydrogels can also be generated using a variety of methods, including PEG cryogels (Dispinar et al. 2012), electrospinning of fibres (Kwon et al. 2017; Matthews et al. 2002; Pham et al. 2006) or alginate hydrogels with engineered microcavities (Zeng et al. 2014). 3D PEG hydrogels fabricated with micro- or macro-pores have been useful to study angiogenesis and vascularisation (Dziubla and Lowman 2004; Oliviero et al. 2012). PEG chains can also be used as porogens to generate hydrogel membranes with controlled permeabilities (Decock et al. 2018).

It will be desirable to develop materials with reversible or dynamically altered properties. This might be facilitated by the development of controllable porogens or by the use of nano- or micro-patterned silk fibres (Xiao et al. 2018) that could mimic native tissue architecture. In particular, engineering ECM topography would be facilitated by recent advances in 3D bioprinting. For example, direct ink writing allows to combine hydrogels, ECM components and cells into complex ‘tissue-mimicking’ constructs on a layer by layer fashion even in the absence of scaffolds (reviewed in (Ji and Guvendiren 2017)). Such systems are currently used to study stem cell differentiation with evident applications in regenerative medicine (Gopinathan and Noh 2018), but incorporating malignant ECM along with stromal or cancer cells in such structures would significantly enhance our palette of tools for understanding the role of ECM in cancer. Further technical developments as well as the incorporation of bioinks derived from different ECM environments such as decellularized tissues (Choudhury et al. 2018) would rapidly improve our control of the architecture, mechanics and biology of fabricated materials, in a precise and reproducible way, paving the way to the design and development of reliable ‘organ-’ or even ‘tumour-’on-a-chip approaches.

### Outlook for the future and translation

Tumorigenesis destabilises normal tissue architecture and thus throws forces in the affected tissue out of balance. Gaining a full understanding of how the different physical and biological aspects of the ECM control cancer cell behaviour, from genome integrity to motility and invasion, will be informative for appreciating what delineates metastatic disease and dormancy. To achieve this formidable task, better tools need to be developed not only to monitor and visualise ECM properties in vivo but also to precisely and controllably model them in vitro*.* Elastography, a method to image collagen density shows great promise for identifying tissue stiffness in biopsies, correlating to disease stage. This has been further expanded to assess viscoelastic properties (Sinkus et al. 2007). However, further progress is required to increase imaging quality and to apply more sophisticated image analysis algorithms to stratify patients and hopefully to predict metastatic spread or disease recurrence. To further understand the involvement of ECM in cancer progression and in the control of quiescent versus proliferative properties of tumour cells, engineered materials with controlled properties, on a reversible and independent manner, are required. This might be facilitated by the use of novel chemicals and the incorporation of full-length native ECM-derived proteins. These could act as scaffolds to present different growth factors or diffusible chemical signals to cells, on a controllable or stress-related way. Fibronectin, for example, has the ability to bind growth factors such as TGF-β or BMP-2 and keep them in a latent form to be presented to cells (Grigoriou et al. 2017). Controllable stretching or degradation of these growth factor–bound fibres might not only change the mechanical properties but also causes release of signalling molecules causing them to present to cells in a physiologically relevant way.

The ultimate aim is to identify therapies that could target cancer cells using an efficient and holistic approach. Understanding which aspects facilitate or restrict cancer spread, how dormant tumorigenic cells are awakened and what are the requirements for successful seeding of a distant secondary tissue will contribute to therapeutic developments. Since chemotherapeutic agents must diffuse into the ECM to access the tumour bulk, ECM topography, confinement and vascularisation are important aspects to consider when designing and testing new agents. Novel engineered microenvironments will prove useful for drug screening allowing more physiologic tests of drug efficiency.

ECM mechanics play key roles in a variety of diseases, so cross-disease studies may offer new insights, such as correlating the effects of fibrosis or arthritis and cancer. It seems that reshaping of the cancer ECM along with common chemotherapeutic strategies might provide promise in the future (Vennin et al. 2018). However, elucidating further how ECM mechanics and architecture shape malignancy will expand both our understanding and therapeutic tools against malignancy.

## Electronic supplementary material

ESM 1 (DOCX 44.5 kb)
