# Supplementary material for: Tissue engineering the cancer microenvironment—challenges and opportunities
Source: Biophys Rev. 2018 Nov 8;10(6):1695–711. doi: 10.1007/s12551-018-0466-8 (PMC6297082; doi:10.1007/s12551-018-0466-8)
Supplement: Supplementary file 1 — (DOCX 44.5 kb) [file 12551_2018_466_MOESM1_ESM.docx]

**Box – Main extracellular proteins directing matrix viscoelasticity**

**Collagen**

Collagens are the most abundant molecules of the ECM with a widespread distribution throughout the human body. They are a large family of triple helical proteins, providing a variety of biomechanical signals. Their triple polypeptide chains containing repeating Gly-X-Y triplets (X and Y often occupied by proline and 4-hydroxyproline respectively) offer increased conformational flexibility, allowing generation of supramolecular complexes and elongated fibrils (Gordon and Hahn, 2010). Collagens provide structural support and elasticity, as they are the main component of stiff tissues, like tendons, bone and skin. Stretching of collagen fibres can contribute to tensile stress, while their non-centrosymmetric nature attributes nonlinear optical susceptibility that can be exploited for development of advanced imaging techniques, such as second harmonic generation (SHG) imaging. In addition, collagens carry various cell adhesion motifs, such as RGD and GFOGER peptides, that allow multiparametric interactions with epithelial cells. Vertebrate collagens can be classified by their functions and domain homology. Briefly, they can be categorised as: fibril-forming, fibril-associated with interrupted triple helices (FACITs), network-forming, transmembrane, endostatin-producing, anchoring fibrils or beaded filament forming collagens. Collagen I can be considered as a collagen prototype and one of the most characterised collagens on both normal and malignant ECM. In particular, collagen I secretion and assembly require a series of posttranslational modifications, including hydroxylation of proline and lysine residues as well as cleavage of N- and C- terminal pro-peptides (Kadler et al., 2007). Collagen I fibres can be further crosslinked between lysine residues by lysyl oxidases (LOX). Collagen deposition and increased LOX activity have been extensively described in cancer progression. This deposition might increase pressure locally around the tumour cells and increase the stiffness of the stroma. High ECM tension might promote bending of collagen fibres and increasing cell traction forces and secretion of matrix remodelling enzymes could lead to collagen degradation allowing cell invasion. It has been speculated that collagen can have both tumour protective and tumour promoting roles (Fang et al., 2014). Elucidating the precise multifaceted functions of the different collagen molecules on cancer cells as well as their role in metastatic progression is necessary in order to understand completely the contribution of these molecules in tumorigenesis.

**Elastin**

Elastin is a glycoprotein composed by precursor tropoelastin monomers that can self-assemble into elastic fibres. It is an important ECM protein offering structural support to tissues and blood vessels. In particular, elastin displays the ability to recover from stretch or contraction, therefore indicating an important role in dictating ECM elastic properties. It is associated mainly with collagens and fibrillin generating durable ECM scaffolds (Baldwin et al., 2013). Single tropoelastin subunits can be crosslinked through layers of fibrillin microfibrils. In addition, crosslinking of lysine residues by members of lysyl oxidase (LOX) family can induce assembly of tropoelastin monomer into insoluble fibres (Noblesse et al., 2004). Elastin has been implicated in tumorigenesis favouring cancer cell growth, invasion and migration (Lapis and Timar, 2002). Considering the role of ECM stiffness in tumour progression, the precise roles of elastin implications in tumorigenesis remain to be revealed.

**Fibronectin**

Fibronectin is a large glycoprotein of 240-270kDa that in tissues is usually incorporated into a fibrillar extracellular matrix. It is assembled as an antiparallel dimer that displays both structural integrity through disulphide bond binding near the C-terminus of its monomers, but also extensive flexibility at the monomer core (Singh et al., 2010). Fibronectin is characterised by the presence of several sites for cell recognition, including RGD motifs the main integrin receptor ligand sites, but also a variety of sites for interacting with other ECM components. Integrin-mediated interactions are important for fibronectin fibril assembly, as soluble fibronectin molecules usually acquire compact conformations and fail to generate fibrillar matrices. Specifically, integrin binding causes conformational alterations to fibronectin molecules exposing self-assembly sites. Force applied by the cells has been shown to promote unfolding of fibronectin molecules and trigger fibrillogenesis (Smith et al., 2007). Due to the biphasic nature of its monomers, fibronectin fibres can offer increased structural support to the ECM but also flexibility, indicating a physiological role in directing matrix viscoelasticity. Usually tumours are characterised by increased fibronectin deposition and fibrillogenesis favouring invasive transformation (Wang and Hielscher, 2017).

**Hyaluronan**

Hyaluronan, or hyaluronic acid (HA), is an extracellular polysaccharide that is found in the ECM of many adult tissues, including mainly soft connective tissues. A unique feature of hyaluronan is that it is the only glycosaminoglycan protein with unsulfated disaccharides and it is not covalently attached to a proteoglycan core protein (Gandhi and Mancera, 2008). It has been indicated that it is the polymer size rather than conformational changes that are important for exerting the biological functions of hyaluronan. Hyaluronan chains are synthesised by HA synthases and degraded by hyaluronidases. This balance between synthesis and degradation in an important regulator of normal tissue HA homeostasis. Loss of this balance has been associated with cancer onset and progression. Not only is the deposition of hyaluronan higher as cancer progresses, but also many tumour-promoting processes have been linked to hyaluronan fragments. In particular, fragmentation of hyaluronan can be caused by tissue stress and reactive oxygen/nitrogen species (ROS/NOS) inducing pro-inflammatory and pro-fibrotic responses. Both cancer cell derived and CAF-derived hyaluronan seems to be important for its deposition in PDAC, associating with a more desmoplastic stroma (Cheng et al., 2013; Kultti et al., 2014). The high molecular weight together with the semi-flexible nature of HA polymers contribute to the increased viscosity of HA solutions. The increased presence of hyaluronan in tumours might therefore help resistance to compressive stress (reviewed in (Stylianopoulos, 2017)). Intriguingly, the ability of naked mole rats to generate hyaluronan of very high molecular mass seems to drive the resistance of these animals to cancer (Tian et al., 2013). Therefore, hyaluronan is on the spotlight for developing ECM targeted therapeutics. Not only it would be possible to increase or decrease mechanical pressure on the tumour cells by altering hyaluronidases levels but also it can be used to tag drugs facilitating uptake by cells (reviewed in (Venning et al., 2015)).

**Laminin**

Laminins are a family of approximately 20 cross- or T-shaped heterotrimeric glycoproteins that have been associated mainly with basement membranes as fabrication of the latter seem to depend on laminins’ self-assembly ability into sheet-like structures (McKee et al., 2007). Laminin has been shown to interact with Collagen IV as well as other ECM molecules but it is also able to bind to cell surface receptors through its different peptide sequences that include RGD peptides, E8 fragments or IKVAV sequences. Laminins have been implicated to essential morphogenesis events of rich in basement membrane tissues including the intestine (reviewed in (Simon-Assmann et al., 1998)) and the vasculature (reviewed in (Kostourou and Papalazarou, 2014)). In tumorigenesis, laminins have implicated to vascular mimicry, a process where cancer cells assemble ECM-rich tubular networks (Seftor et al., 2001). In addition, loss of laminin has been suggested to interfere with the polarisation of luminal breast epithelial cells promoting carcinogenesis (Gudjonsson et al., 2002). Interestingly, laminin mediated signalling and ECM stiffness have been inversely correlated for functional mammary epithelial cell differentiation indicating the importance of those pathways in breast tumorigenesis (Alcaraz et al., 2008). This observation arises many questions regarding the role of laminins in tumorigenesis. How do the different laminin isoforms interact with cancer cells or other extracellular molecules to define ECM mechanical properties and how their expression alters during malignancy progression *in vivo* remains to be identified. Considering that cancer cells have to breach their basement membrane in order to invade, does loss of laminins facilitates this process by rupturing the normal basement membrane or generating pores increasing invasive capacity of cancer cells? If that was true, do laminins have a tumour suppressive role and could they be therapeutically utilised to normalise malignant stroma and vasculature?

**Tenascins**

Tenascins can be found in the ECM of tissues as five different apparitions, TN-C, TN-R, TN-W, TN-X and TN-Y (Tucker and Chiquet-Ehrismann, 2009). In particular, tenascin C has been majorly implicated with the ECM of desmoplastic tumours as well as metastatic niches favouring aggressiveness. Tenascin C is a hexameric protein where two trimers of tenascin chains are joined to form a multimodular protein having the ability to interact with a plethora of other ECM proteins, mainly fibronectin and collagens (reviewed in (Midwood et al., 2016)). Apart from interfering to fibronectin signalling to cells is also able to bind directly to integrins modulating cell adhesions, containing its integrin-binding RGD motifs (Tucker and Chiquet-Ehrismann, 2015). Tenascin C has been linked to regulate the mechanical activity of the ECM. By interacting with other ECM components has been shown to offer structural support defining ECM stiffness. In addition, its expression has been shown to be induced by mechanical stress indicating a positive loop (Tucker and Chiquet-Ehrismann, 2009). Specifically, it has been shown to orchestrate fibrotic responses in pathophysiology (Bhattacharyya et al., 2016). Furthermore, it seems to favour cancer progression through a multiparametric role and a variety of functions, including disruption of actin fibres and Wnt signalling activation (Saupe et al., 2013), angiogenesis (Langlois et al., 2014) and EMT (Shao et al., 2015). It seems also important for the lung colonisation of metastatic breast cancer cells (Oskarsson et al., 2011). Recently, Tenascin C has been linked to modifying ECM stiffness and mechanosignalling in glioblastoma (Miroshnikova et al., 2016). Further understanding of the biomechanical functions of tenascin proteins is required to understand their role in metastatic dissemination and dormancy awakening.

References:

Alcaraz, J., Xu, R., Mori, H., Nelson, C.M., Mroue, R., Spencer, V.A., Brownfield, D., Radisky, D.C., Bustamante, C., and Bissell, M.J. (2008). Laminin and biomimetic extracellular elasticity enhance functional differentiation in mammary epithelia. EMBO J *27*, 2829-2838.

Baldwin, A.K., Simpson, A., Steer, R., Cain, S.A., and Kielty, C.M. (2013). Elastic fibres in health and disease. Expert Rev Mol Med *15*, e8.

Bhattacharyya, S., Wang, W., Morales-Nebreda, L., Feng, G., Wu, M., Zhou, X., Lafyatis, R., Lee, J., Hinchcliff, M., Feghali-Bostwick, C.*, et al.* (2016). Tenascin-C drives persistence of organ fibrosis. Nat Commun *7*, 11703.

Cheng, X.B., Sato, N., Kohi, S., and Yamaguchi, K. (2013). Prognostic impact of hyaluronan and its regulators in pancreatic ductal adenocarcinoma. PLoS One *8*, e80765.

Fang, M., Yuan, J., Peng, C., and Li, Y. (2014). Collagen as a double-edged sword in tumor progression. Tumour Biol *35*, 2871-2882.

Gandhi, N.S., and Mancera, R.L. (2008). The structure of glycosaminoglycans and their interactions with proteins. Chem Biol Drug Des *72*, 455-482.

Gordon, M.K., and Hahn, R.A. (2010). Collagens. Cell Tissue Res *339*, 247-257.

Gudjonsson, T., Ronnov-Jessen, L., Villadsen, R., Rank, F., Bissell, M.J., and Petersen, O.W. (2002). Normal and tumor-derived myoepithelial cells differ in their ability to interact with luminal breast epithelial cells for polarity and basement membrane deposition. J Cell Sci *115*, 39-50.

Kadler, K.E., Baldock, C., Bella, J., and Boot-Handford, R.P. (2007). Collagens at a glance. J Cell Sci *120*, 1955-1958.

Kostourou, V., and Papalazarou, V. (2014). Non-collagenous ECM proteins in blood vessel morphogenesis and cancer. Biochim Biophys Acta *1840*, 2403-2413.

Kultti, A., Zhao, C., Singha, N.C., Zimmerman, S., Osgood, R.J., Symons, R., Jiang, P., Li, X., Thompson, C.B., Infante, J.R.*, et al.* (2014). Accumulation of extracellular hyaluronan by hyaluronan synthase 3 promotes tumor growth and modulates the pancreatic cancer microenvironment. Biomed Res Int *2014*, 817613.

Langlois, B., Saupe, F., Rupp, T., Arnold, C., van der Heyden, M., Orend, G., and Hussenet, T. (2014). AngioMatrix, a signature of the tumor angiogenic switch-specific matrisome, correlates with poor prognosis for glioma and colorectal cancer patients. Oncotarget *5*, 10529-10545.

Lapis, K., and Timar, J. (2002). Role of elastin-matrix interactions in tumor progression. Semin Cancer Biol *12*, 209-217.

McKee, K.K., Harrison, D., Capizzi, S., and Yurchenco, P.D. (2007). Role of laminin terminal globular domains in basement membrane assembly. J Biol Chem *282*, 21437-21447.

Midwood, K.S., Chiquet, M., Tucker, R.P., and Orend, G. (2016). Tenascin-C at a glance. J Cell Sci *129*, 4321-4327.

Miroshnikova, Y.A., Mouw, J.K., Barnes, J.M., Pickup, M.W., Lakins, J.N., Kim, Y., Lobo, K., Persson, A.I., Reis, G.F., McKnight, T.R.*, et al.* (2016). Tissue mechanics promote IDH1-dependent HIF1alpha-tenascin C feedback to regulate glioblastoma aggression. Nat Cell Biol *18*, 1336-1345.

Noblesse, E., Cenizo, V., Bouez, C., Borel, A., Gleyzal, C., Peyrol, S., Jacob, M.P., Sommer, P., and Damour, O. (2004). Lysyl oxidase-like and lysyl oxidase are present in the dermis and epidermis of a skin equivalent and in human skin and are associated to elastic fibers. J Invest Dermatol *122*, 621-630.

Oskarsson, T., Acharyya, S., Zhang, X.H., Vanharanta, S., Tavazoie, S.F., Morris, P.G., Downey, R.J., Manova-Todorova, K., Brogi, E., and Massague, J. (2011). Breast cancer cells produce tenascin C as a metastatic niche component to colonize the lungs. Nat Med *17*, 867-874.

Saupe, F., Schwenzer, A., Jia, Y., Gasser, I., Spenle, C., Langlois, B., Kammerer, M., Lefebvre, O., Hlushchuk, R., Rupp, T.*, et al.* (2013). Tenascin-C downregulates wnt inhibitor dickkopf-1, promoting tumorigenesis in a neuroendocrine tumor model. Cell Rep *5*, 482-492.

Seftor, R.E., Seftor, E.A., Koshikawa, N., Meltzer, P.S., Gardner, L.M., Bilban, M., Stetler-Stevenson, W.G., Quaranta, V., and Hendrix, M.J. (2001). Cooperative interactions of laminin 5 gamma2 chain, matrix metalloproteinase-2, and membrane type-1-matrix/metalloproteinase are required for mimicry of embryonic vasculogenesis by aggressive melanoma. Cancer Res *61*, 6322-6327.

Shao, H., Kirkwood, J.M., and Wells, A. (2015). Tenascin-C Signaling in melanoma. Cell Adh Migr *9*, 125-130.

Simon-Assmann, P., Lefebvre, O., Bellissent-Waydelich, A., Olsen, J., Orian-Rousseau, V., and De Arcangelis, A. (1998). The laminins: role in intestinal morphogenesis and differentiation. Ann N Y Acad Sci *859*, 46-64.

Singh, P., Carraher, C., and Schwarzbauer, J.E. (2010). Assembly of fibronectin extracellular matrix. Annu Rev Cell Dev Biol *26*, 397-419.

Smith, M.L., Gourdon, D., Little, W.C., Kubow, K.E., Eguiluz, R.A., Luna-Morris, S., and Vogel, V. (2007). Force-induced unfolding of fibronectin in the extracellular matrix of living cells. PLoS Biol *5*, e268.

Stylianopoulos, T. (2017). The Solid Mechanics of Cancer and Strategies for Improved Therapy. J Biomech Eng *139*.

Tian, X., Azpurua, J., Hine, C., Vaidya, A., Myakishev-Rempel, M., Ablaeva, J., Mao, Z., Nevo, E., Gorbunova, V., and Seluanov, A. (2013). High-molecular-mass hyaluronan mediates the cancer resistance of the naked mole rat. Nature *499*, 346-349.

Tucker, R.P., and Chiquet-Ehrismann, R. (2009). The regulation of tenascin expression by tissue microenvironments. Biochim Biophys Acta *1793*, 888-892.

Tucker, R.P., and Chiquet-Ehrismann, R. (2015). Tenascin-C: Its functions as an integrin ligand. Int J Biochem Cell Biol *65*, 165-168.

Venning, F.A., Wullkopf, L., and Erler, J.T. (2015). Targeting ECM Disrupts Cancer Progression. Front Oncol *5*, 224.

Wang, J.P., and Hielscher, A. (2017). Fibronectin: How Its Aberrant Expression in Tumors May Improve Therapeutic Targeting. J Cancer *8*, 674-682.
